# Development and evaluation of recombinant GRA8 protein for the serodiagnosis of *Toxoplasma gondii* infection in goats

**DOI:** 10.1186/s12917-020-02719-3

**Published:** 2021-01-09

**Authors:** Charoonluk Jirapattharasate, Ruenruetai Udonsom, Apichai Prachasuphap, Kodcharad Jongpitisub, Panadda Dhepakson

**Affiliations:** 1 Department of Preclinic and Applied Animal Science, Faculty of Veterinary Science, Mahidol University, 999 Phutthamonthon sai 4 Rd, Salaya, Nakhonpathom, 73170 Thailand; 2 Department of Protozoology, Faculty of Tropical Medicine, Mahidol University, 420/6 Ratchawithi Road, Ratchathewi, Bangkok, 10400 Thailand; 3 Department of Medical Sciences, Medical Life Sciences Institute, 88/7 Tiwanon Road, Talad Kwan Subdistrict, Muang District, Nonthaburi, 11000 Thailand

**Keywords:** *Toxoplasma gondii*, GRA8, Serodiagnosis, Goat, Gene synthesis

## Abstract

**Background:**

The development of sensitive and specific methods for detecting *Toxoplasma gondii* infection is critical for preventing and controlling toxoplasmosis in humans and other animals. Recently, various recombinant proteins have been used in serological tests for diagnosing toxoplasmosis. The production of these antigens is associated with live tachyzoites obtained from cell cultures or laboratory animals for genomic extraction to amplify target genes. Synthetic genes have gained a key role in recombinant protein production. For the first time, we demonstrated the production of the recombinant protein of the *T. gondii* dense granular antigen 8 (TgGRA8) gene based on commercial gene synthesis. Recombinant TgGRA8 plasmids were successfully expressed in an *Escherichia coli* system. The recombinant protein was affinity-purified and characterized via sodium dodecyl sulfate-polyacrylamide gel electrophoresis and Western blotting. Furthermore, the diagnostic potential of the recombinant protein was assessed using 306 field serum samples from goats via indirect enzyme-linked immunosorbent assay (iELISA) and the latex agglutination test (LAT).

**Results:**

Western blotting using known positive serum samples from goats identified a single antigen at the expected molecular weight of TgGRA8 (27 kDa). iELISA illustrated that 15.40% of goat samples were positive for *T. gondii*-specific IgG antibodies. In addition, TgGRA8 provided high sensitivity and specificity, with significant concordance (91.83) and kappa values (0.69) compared with the results obtained using LAT.

**Conclusion:**

Our findings highlight the production of a recombinant protein from a synthetic TgGRA8 gene and the ability to detect *T. gondii* infection in field samples. The sensitivity and specificity of TgGRA8 demonstrated that this protein could be a good serological marker for detecting specific IgG in goat sera.

## Background

Toxoplasmosis is caused by the protozoan parasite *Toxoplasma gondii*, and this infection is widespread in humans and animals, occurring in approximately 25–30% of the human population [1]. Most people infected with *T. gondii* are asymptomatic; however, fatal encephalitis caused by this protozoan can be observed in immunocompromised patients [2]. Infection in humans and animals, as the intermediate hosts, occurs mainly through the ingestion of raw or undercooked meat containing viable tissue cysts or through exposure to soil, food, or water contaminated with oocysts passed in the feces of infected cats or other felines [3]. Normally, farm animals display no clinical symptoms, although *T. gondii* infection may induce abortion, leading to reproductive losses in the livestock industry [4].

Serological methods play a major role in the diagnosis of *T. gondii* infection in humans and animals [5, 6]. Several serological tests have been developed using either live tachyzoites or native soluble antigens; however, they are expensive, laborious, and nonspecific [7]. Recently, recombinant *T. gondii* antigens were identified as good candidates for replacing native antigens because they are easily produced in large volumes using standardized methods [8, 9]. Dense granule antigens (GRAs) of *T. gondii* are secreted in the parasitophorous vacuole (PV), and they are involved in survival and virulence of the parasite [10]. Several studies demonstrated the diagnostic potential of numerous GRAs such as GRA2 [11], GRA5 [12], GRA6 [13], and GRA7 [14, 15]. GRA8 is a 38-kDa praline-rich (24%) protein that is released from PVs shortly after invasion. GRA8 is a 269-amino acid polypeptide with a terminal signal peptide, three degenerate proline-rich repeats in the central region, and a potential transmembrane domain near the carboxy-terminal region [16]. Previous studies used the recombinant GRA8 protein in specific IgM and IgG enzyme-linked immunosorbent assay (ELISA) in humans [17–19]. However, little information is available concerning the use of recombinant GRA8 protein-based ELISA for the serodiagnosis of *T. gondii* infection in animals.

Regarding recombinant protein production in *T. gondii*, the mRNA of target genes is extracted from live tachyzoites and recombinant plasmid is transformed to bacteria for protein expression. However, the transfer of gene sequences between organisms may not be successful, leading to low level protein expression because of differences in codon usage [20, 21]. Recently, synthetic gene synthesis has been used to design and create genes without an existing DNA template [22]. In addition, gene synthesis tools do not require access to a pathogen, thus preventing the exposure of research staff to harmful living parasites [23]. In this study, we used a synthesized *T. gondii* GRA8 gene (designated TgGRA8) as a DNA template for recombinant protein production. Furthermore, the purified protein was used in specific IgG indirect ELISA (iELISA) in the diagnosis of *T. gondii* infection using goat sera. The latex agglutination test (LAT) was used to validate the detection system in this study.

## Results

### Construction of the recombinant TgGRA8 plasmids

**Fig. 1:**
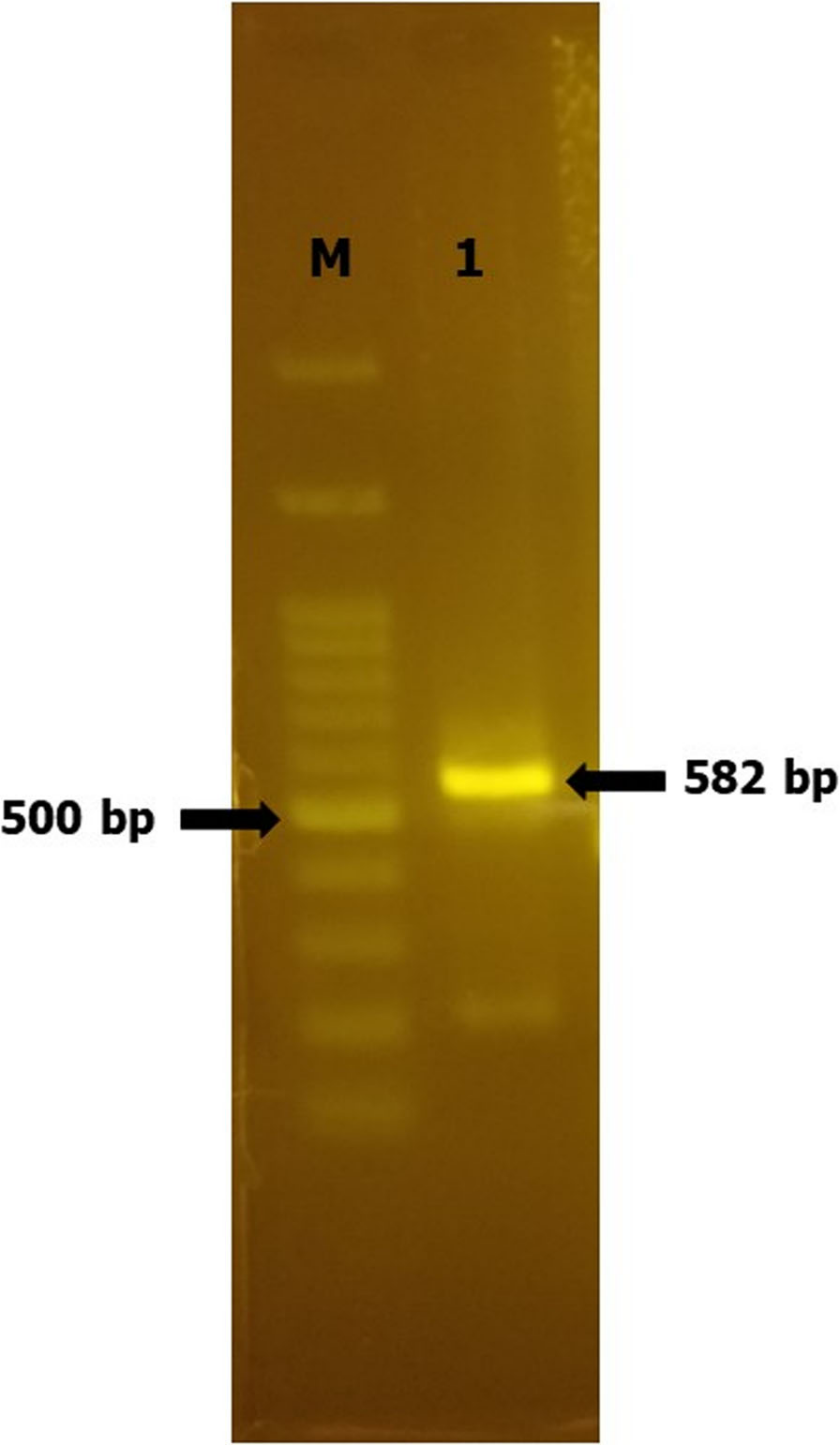
Screening of the PCR product of the TgGRA8 gene. Lane M, 100-bp DNA ladder; Lane 1, 582-bp PCR product after digestion with *Nde*I and *Age*I

The 582-bp GRA8 gene was PCR-amplified from synthetic TgGRA8 (Fig. 1). The PCR product was purified and double digested with NdeI and AgeI. The digested product (25 ng/ml) was used for ligation. *E. coli* DH5α-competent cells were transformed with recombinant pET-21a vectors and cultured with 2XTY agar containing ampicillin. Positive colonies were identified by colony PCR. Sequence analysis of the clone revealed 100% homology with the sequence of recombinant TgGRA8.

### Purification and Western blot analysis of expressed protein

**Fig. 2:**
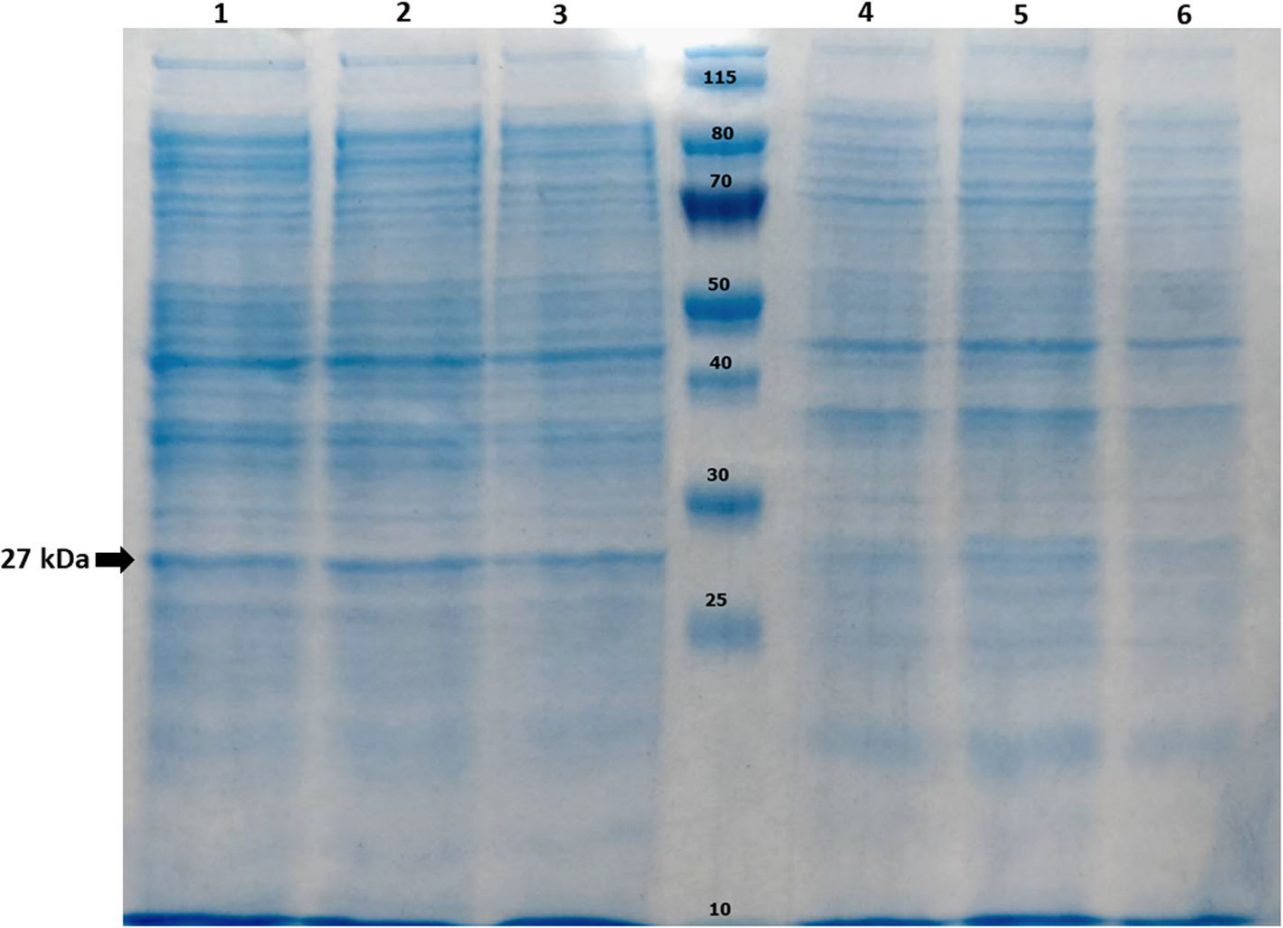
Sodium dodecyl sulfate-polyacrylamide gel electrophoresis analysis on the optimized expression of TgGRA8 in *E. coli* strain Rosetta (DE3), Coomassie blue stained. Lane M: protein molecular weight marker. Lane 1 to 3: pellet fractions of cells grown at 20 °C after induction with 1.0 mM IPTG (4,8 h and overnight). Lane 4 to 6: uninduced total cell lysate of *E. coli* strain Rosetta (DE3)-pET21a-TgGRA8 (4,8 h and overnight)

**Fig. 3:**
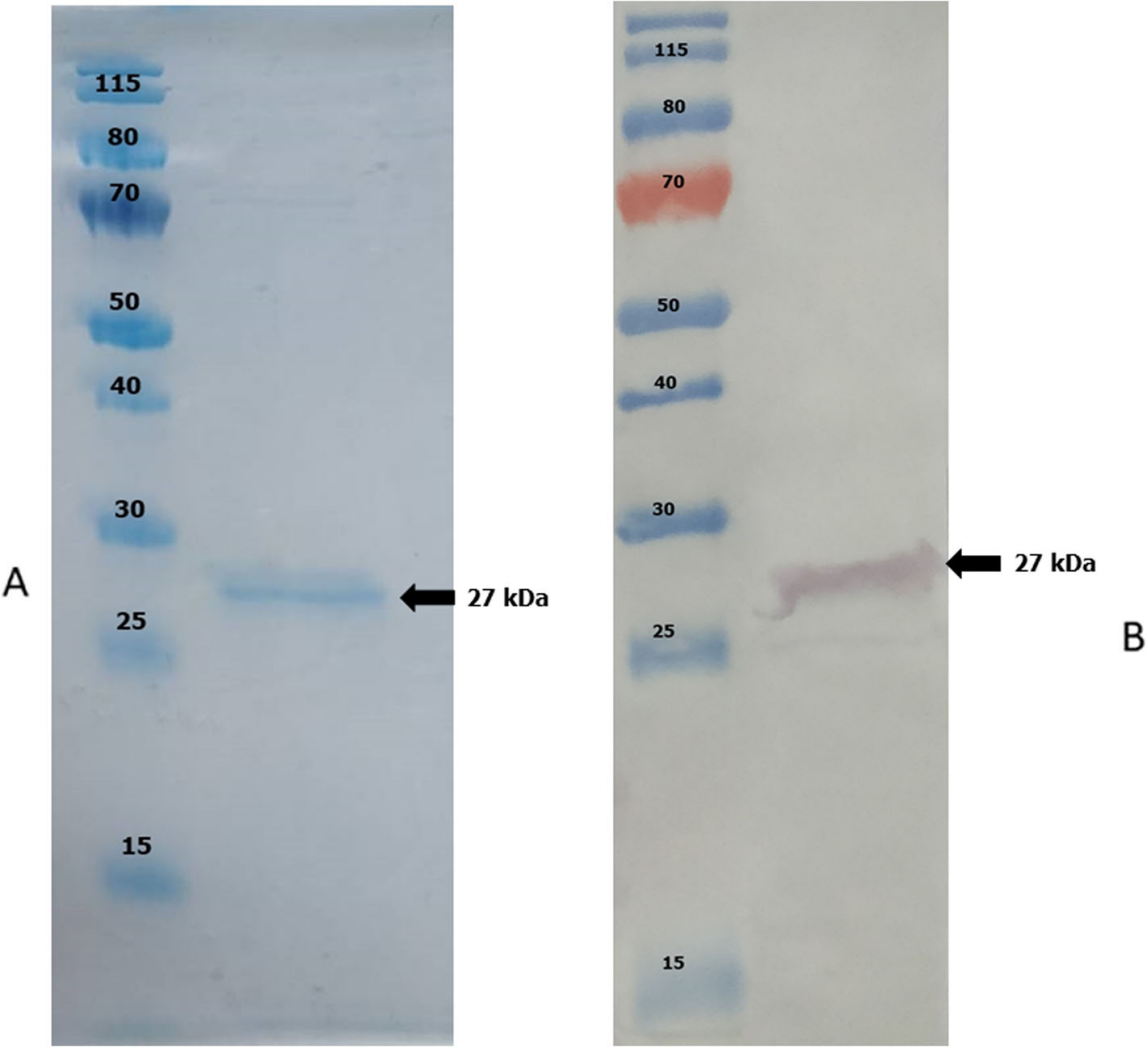
**a** Sodium dodecyl sulfate-polyacrylamide gel electrophoresis analysis of expression of recombinant *Toxoplasma gondii* dense granular antigen 8 (TgGRA8) protein. Lane M, protein molecular weight marker; Lane 1, the soluble recombinant TgGRA8 protein was purified using anti-FLAG tag affinity resin. **b** Western blot analysis of purified recombinant TgGRA8. Lane M, protein molecular weight marker; Lane 1, the purified 27-kDa TgGRA8 protein was detected using an anti-FLAG tag antibody

Production of recombinant TgGRA8 was optimized by altering various incubation periods, and expression levels were analyzed by SDS-PAGE as shown in Fig. 2. A 27 kDa band was observed in the induced bacteria. Expression of this protein increased up to 2 h after induction and remained constant after overnight. To confirm the protein expression, the induced bacteria exhibited a protein expression band of 27 kDa in size after purification using anti-FLAG tag affinity resin (Fig. 3a).

**Fig. 4:**
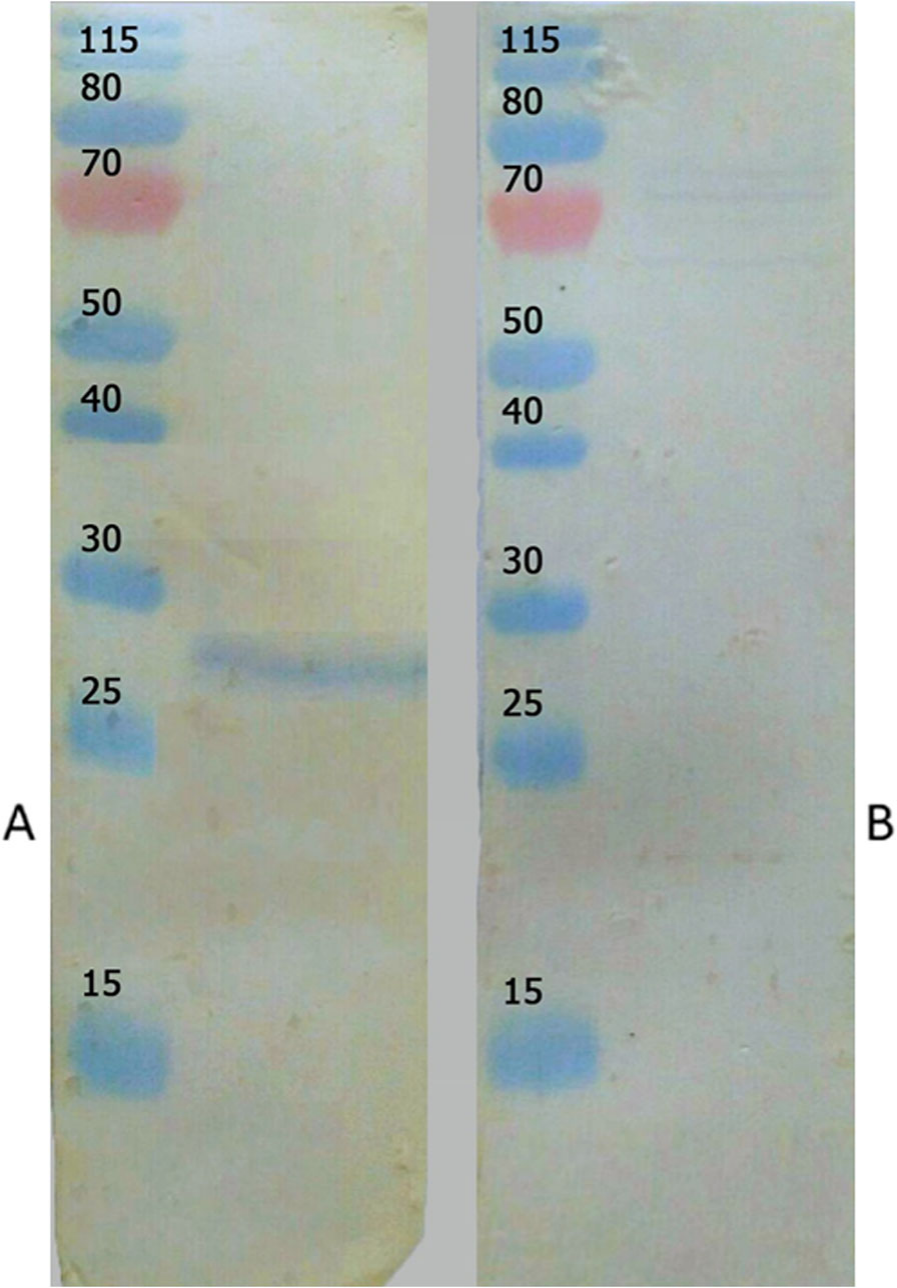
Western blot analysis. Purified proteins were separated via 12% sodium dodecyl sulfate-polyacrylamide gel electrophoresis, transferred to nitrocellulose membranes, and then probed with known positive and negative goat sera. **a** Lane M, protein molecular weight marker; Lane 1, strong reactivity with known positive serum; **b** Lane M, protein molecular weight marker; Lane 1, result for negative serum

The purified protein was analyzed by Western blotting using peroxidase-conjugated anti-FLAG tag antibody (GenScript, USA) diluted 1:1000 in blocking buffer. The result illustrated that the TgGRA8 fusion protein was specifically recognized by anti-FLAG tag antibody (Fig. 3b). However, the specific band size was slightly larger than the estimated size of 22.55 kDa (amino acids 24–217 plus the 2X FLAG tag). The concentration of the protein was measured as 1.26 mg/ml by BSA assay (Pierce Biotechnology, Inc., USA). The specific reactivity and purity of TgGRA8 was checked using known positive and negative serum samples from goats. Western blotting revealed that the TgGRA8 fusion protein was recognized by the known positive serum (Fig. 4).

### Confirmation of TgGRA8 protein

The identity of protein expressed and purified recombinant protein was confirmed by mass spectrometry (MS) analysis. The partial sequence of TgGRA8 in this study shared 98.95–100% identities with database sequences (XP002369526, KFG46645, RQX68523 and AAD55381). Therefore, we confirmed that our expressed recombinant protein was *T. gondii* GRA8.

### Evaluation of the serodiagnostic potential of recombinant TgGRA8 by indirect ELISA (iELISA)

**Fig. 5:**
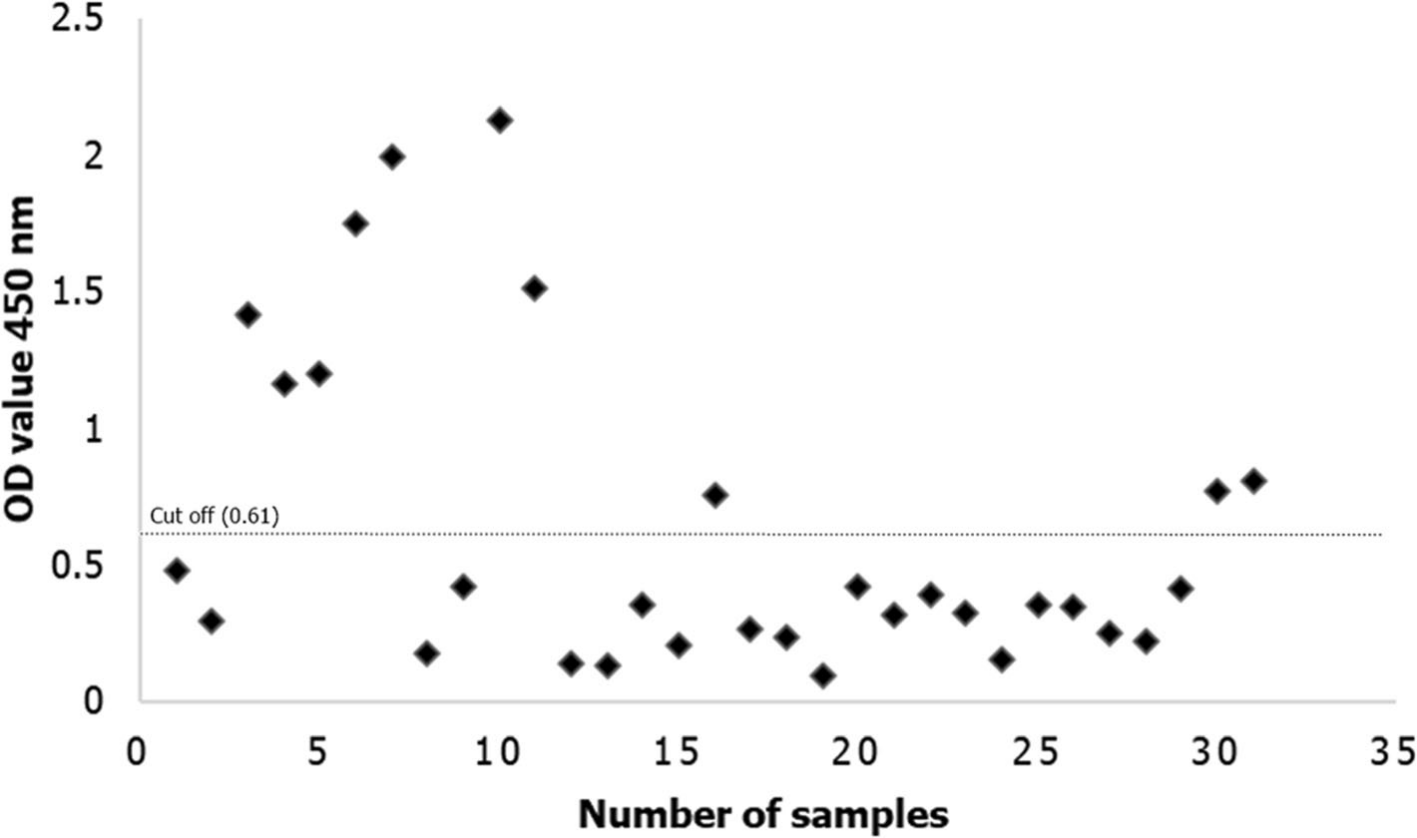
Antibody response to *Toxoplasma gondii* in field sera from goats using recombinant *T. gondii* dense granular antigen 8 protein-based indirect enzyme-linked immunosorbent assays

The serodiagnotic potential of recombinant TgGRA8-iELISA was evaluated for its potential utility in serological testing using known positive (*N* = 10) and negative (*N* = 21) goat sera. The cut-off value was calculated as the average OD_450_ plus three standard deviations of standard *T. gondii*-negative control goat sera. The cut-off value for goats in this study was determined as 0.61 (Fig. 5). Base on recombinant TgGRA8-iELISA, 15.35% goat sera samples were positive for *T. gondii*-specific IgG antibodies.

### Comparison of iELISA and LAT

The diagnostic performance of recombinant TgGRA8-iELISA was evaluated with reference to LAT [14]. The sensitivity and specificity of the recombinant protein and kappa values at 95% confidence interval (95% CI) were calculated. The seropositivity rate of goat samples in LAT was 17.0%. The sensitivity and specificity of LAT for the recombinant protein were 71.1 and 96.0%, respectively. Substantial agreement between the two methods was indicated by κ = 0.69 (Table 1).

**Table 1:** Comparison of LAT and TgGRA8 recombinant protein-based iELISA for the detection IgG antibodies against *Toxoplasma gondii* infection

TgGRA8 iELISA	LAT			Sensitivity (95% CI)	Specificity (95% CI)	Kappa value
	Positive	Negative	Total			
Positive	37	10	47	71.17	96.06	0.69
Negative	15	244	259	(56.72–82.45)	(92.65–97.98)	
Total	52	254	306			

*LAT* Latex agglutination test, *TgGRA8 T. gondii* dense granular antigen 8, *iELISA* Indirect enzyme-linked immunosorbent assay, *CI* Confidence interval

## Discussion

Recently, recombinant DNA technology and synthetic DNA have played important roles in high-quality recombinant antigenic protein production for the serological diagnosis of *T. gondii* infection. Several recombinant proteins have been produced and applied for the detection of *T. gondii* infection. These proteins include rhoptry proteins, matrix proteins, microneme proteins, surface antigens, and GRAs [8]. Among them, the GRA proteins have been considered potential diagnostic antigens and have been used to differentiate the stages of infection [24]. Generally, the method of recombinant protein production requires cDNA extracted from live pathogens as a template to amplify target genes. However, unsuccessful recombinant protein production using natural gene sequences, including no or low expression, inclusion body formation, and protein inactivity, has been described [25]. To overcome these problems, we demonstrated the production of recombinant protein from a synthetic TgGRA8 gene and tested the immunodiagnostic potential of the produced protein via iELISA.

Although codon optimization was used to optimize and enhance protein expression in the present study, we failed to produce the recombinant protein using the full-length TgGRA8 gene. Previous studies described a transmembrane region of the GRA8 gene encoding amino acids 223–242 using bioinformatic prediction [16] and reported that the region can affect host cell growth and decrease protein yield [26]. Therefore, we selected the specific region of the TgGRA8 protein based on the prediction of transmembrane helices in proteins using an online program (http://www.cbs.dtu.dk/services/TMHMM/). After removing the transmembrane region, the gene fragment encoding amino acids 24–217 was used to express the protein, and a specific 27-kDa band was observed on SDS-PAGE. Our result was similar to that of Babaie et al. [18], who designed and expressed a recombinant protein from the GRA8 gene fragment corresponding to amino acids 23–169. However, a difference in size between the apparent band and the calculated molecular weight of TgGRA8 (22.55 kDa) was observed in the present study. The predicted protein encoded by the TgGRA8 gene featured high proline content (54 amino acids). The presence of excessive proline residues in proteins causes structural rigidity in the primary sequence, thereby decreasing the electrophoretic mobility [16]. Regarding the yield of TgGRA8, our production gained lower yields than the method of Babaie and colleague [18]. Therefore, the attempt of using codon-optimisation DNA is not considered advantageous for recombinant GRA8 expression.

Numerous GRA proteins, both single and combinations of proteins, have been applied for the serodetection of animal toxoplasmosis. In cats, a single GRA7 recombinant protein [27] and a mixture of recombinant GRA2, GRA6, GRA7, and GRA15 [28] were used to determine the prevalence of *T. gondii* infection in China and Japan, respectively. Moreover, recombinant GRA7 protein-based ELISA has been used in seroprevalence studies of farm animals in Egypt [29] and Thailand [30]. To date, only one study describing the use of TgGRA8 together with recombinant GRA7 to detect specific IgG antibodies against the parasite was published in domestic turkeys [31].

The potential utility of recombinant TgGRA8 protein in serodiagnosis was assessed using known positive and negative goat sera. The result of Western blotting indicated that the protein is a potential marker for detecting *T. gondii* infection in goats. A previous immunochemical evaluation of TgGRA8 using ELISA recorded high reactivity for the recombinant protein using human sera [19], in line with the present result. A possible explanation for the high OD in this study could be the unspecific epitopes of this antigen in the amino-terminal region [32].

The infection rate in our study was lower than that of 27.9% in a previous report on goats in Satun province, Thailand [33]. The difference of the seroprevalence rate may be attributable to the use of different serological diagnosis techniques (iELISA and LAT) and different sampling areas. The sensitivity and specificity obtained using recombinant TgGRA8 in this study indicated that the recombinant protein could be used as an antigen for serological tests of *T. gondii*. However, the use of recombinant proteins for the serodetection of animal toxoplasmosis may be affected by the immune system in different animal species. Therefore, the antibody response in various animals and the epitope structures of recombinant TgGRA8 should be confirmed.

## Conclusion

Our study produced a recombinant protein from a synthetic TgGRA8 gene. The sensitivity and specificity of TgGRA8 demonstrated that this protein could be a good serological marker for detecting specific IgG in goat sera. Commercial gene synthesis is an alternative tool to support recombinant protein expression in the absence of pathogen access.

## Methods

### Gene synthesis of TgGRA8

The complete GRA8 coding sequence (accession number: TGME49_054720) was obtained from an online database (http://ToxoDB.org). The TgGRA8 sequence consists of 810 nucleotides that encode a 269-amino acid protein. A signal peptide (SPs) of GRA8 was determined using online program, SignalP 4.1 (http://www.cbs.dtu.dk/services/SignalP/). The results showed that small fragments of amino acids 1–23 were expressed as a signal sequence. Therefore, encoding amino acid 24–269 was constructed and inserted into a pET-21a vector using *Nde*I and *Xho*I as the cloning sites (General Biosystems, USA).

### Construction of recombinant TgGRA8

**Fig. 6:**
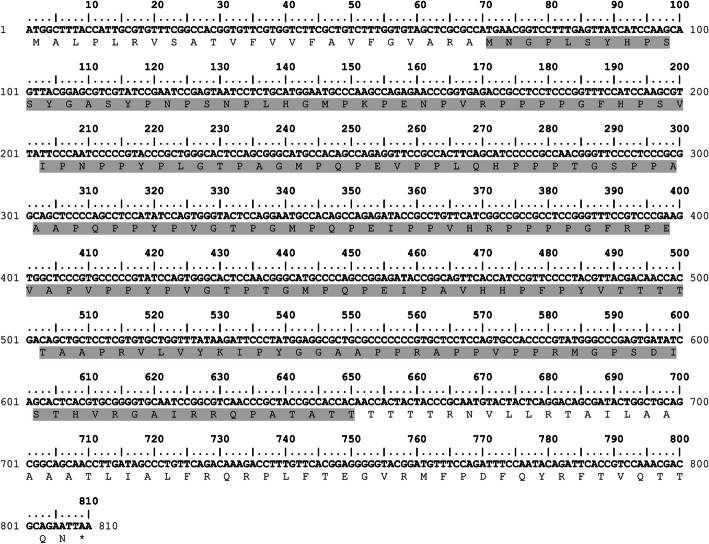
The complete nucleotide and amino acid sequence of *T. gondii* GRA8 (accession number: TGME49_054720). The expression region of GRA8 in this study is delineated by shading (encoding a 194-residual peptide)

The potential transmembrane regions (TMs) of TgGRA8 were predicted by online server (http://www.cbs.dtu.dk/services/TMHMM/). The encoding amino acids 218–269 was transmembrane region. Therefore, only an antigenic fragment of recombinant TgGRA8 encoding amino acids 24–217 was PCR-amplified (Fig. 6). The primers used for amplification of the sequence by PCR was T7 promoter-(FW), 5′-TAA TACG ACT CAC TAT AG-3′ (New England Biolabs, UK); and TgGRA8-RW, 5′-AGT acc ggt GGT GGC GGT TGC CGG CTG-3′. The reverse primer was designed to contain the *Age*I restriction site. PCR was performed using PCR Q5® High-Fidelity DNA Polymerase (New England Biolabs) using the following program: 98 °C for 1 min, followed by 30 cycles of 98 °C for 10 s, 58 °C for 20 s, and 72 °C for 20 s, and final extension at 72 °C for 2 min.

The PCR amplicon was digested using *Nde*I and *Age*I. After digestion, the PCR product was ligated into the modified pET-21a vector harboring a C-terminal fusion protein linker (GGGS) and 2X FLAG tag (DYKDDDDKDYKDDDDK) (General Biosystems, USA) and transformed into *Escherichia coli* DH5α-competent cells.

Ten colonies were selected and expanded in overnight cultures, and DNA was extracted using a QIAprep Spin Miniprep Kit (Qiagen, Germany). The insert of TgGRA8 in the purified plasmid was sequenced using a Dye Terminator Cycle Sequencing Kit (Applied Biosystems, USA) and the 3500xL genetic analyzer (Applied Biosystems). The TgGRA8 sequences were determined using Bioedit version 7.2.5 (Tom Hall Ibis Biosciences, USA).

### Expression of TgGRA8

The recombinant TgGRA8 plasmids were transformed into *E. coli* strain Rosetta (DE3) cells and cultivated in 2XTY supplemented with 1% glucose and 200 ng/ml ampicillin at 37 °C with shaking at 200 rpm. *E. coli* carrying recombinant TgGRA8 was measured at an optimal density at 600 nm (OD600) of 0.5 and induced with isopropyl-β-D-thiogalactopyranoside at a final concentration of 1 mM 20 °C for various incubation periods (2, 4 and overnight) with shaking at 250 rpm. The induced bacteria were harvested via centrifugation at 4400×g for 20 min at 4 °C, and the bacterial pellet was resuspended in 20 ml of pre-chilled lysis buffer (150 mM NaCl, 50 mM Tris-HCL [pH 9.5], 1% Triton X-100, 1 mM EDTA [pH 8.0], and 1% NP 40) and then incubated at 4 °C for 30 min. After incubation, the bacterial cells were lysed via sonication on ice for 10 min, and 1 ml of 1× Protease Inhibitor Cocktail was added (Promega, USA). TgGRA8 expression was analyzed using 12% sodium dodecyl sulfate-polyacrylamide gel electrophoresis (SDS-PAGE).

### Protein purification

Anti-DYKDDDDK G1 affinity resin (GenScript, USA) was used for protein purification. The debris was centrifuged at 10,000×g for 30 min at 4 °C, after which the supernatant was transferred to a clean tube. The resin suspension (600 μl) was loaded into an empty gravity flow column (Bio-Rad, USA) and washed with Tris-buffered saline (50 mM Tris-HCl, 150 mM NaCl, pH 7.4). Protein was eluted from the resin using alkaline elution buffer (0.1 M Tris, 0.5 M NaCl, pH 12.0) and neutralized with 1 M HCl. The protein concentration was measured using NanoDrop ND-1000 UV/Vis spectrophotometer (Thermo Fisher Scientific, USA).

The eluted fractions were dialyzed using SnakeSkin Dialysis Tubing, 10 kDa cut-off (Thermo Fisher Scientific) against phosphate-buffered saline (PBS, pH 7.2) at 4 °C. The debris formed during dialysis was removed via centrifugation at 10,000×g for 5 min at 4 °C, and the concentration of the purified recombinant protein was assayed using both SDS-PAGE and a Coomassie protein assay reagent kit using BSA according to the manufacturer’s protocol (Pierce Biotechnology, Inc., USA).

To confirm the sequence of the recombinant protein, the mass spectrometry (MS) analysis was carried out by Proteomics Services Center, Faculty of Medical Technology, Mahidol University.

### Western blotting

Five micrograms of recombinant TgGRA8 was resolved by 12% SDS-PAGE and then electrotransferred (Trans-blot, Bio-Rad) onto a nitrocellulose membrane (Millipore, USA). The membrane was washed three times with PBS, blocked with 5% skim milk, and then incubated at 37 °C for 1 h with constant shaking. After incubation, the membrane was washed three times with PBS containing 0.01% Tween 20 (PBS-T) and rinsed with PBS. The TgGRA8 protein in nitrocellulose membrane was probed using antibody or known reference positive and negative goat sera (diluted 1:250 in 5% skim milk) kept in our laboratory and incubated at 37 °C for 1 h with constant shaking. The monoclonal antibody (mAb) against Flag-tag (GenScript, USA) was diluted 1:1000, while polyclonal mouse anti-goat immunoglobulin/HRP (Dako, Denmark) was diluted 1:2000 in blocking buffer. After incubation, the membrane was washed three times with PBS-T. The protein band was developed according to peroxidase activity using 3,3′,5,5′-tetramethylbenzidine (KPL, Gaithersburg, MD, USA).

### Goat serum samples

The process of sample collection was reviewed and approved by the Animal Care and Use Committee of the Faculty of Veterinary Science, Mahidol University, Thailand (Approval No. MUVS-2018-03-09). A total of 306 serum samples were obtained from a goat farm in Kanchanaburi province, Thailand. The goats were restrained by holding the base of the horn and blood was collected from the jugular vein and immediately transferred into 10 ml vacuum blood tubes without anticoagulant. The animals were not allowed returned to their cage until complete hemostasis has been achieved. All blood samples were kept in cooled box with ice pack and sent to the laboratory at Faculty of Veterinary Science, Mahidol University. The sera were separated after sedimentation of blood cells and stored at − 20 °C until examination.

### IgG iELISA

Purified recombinant TgGRA8 was diluted at a final concentration of 0.1 μg/ml in coating buffer (50 mM bicarbonate, pH 9.6) and added to separate wells of the ELISA plates (Nunc, Denmark). The coated plates were incubated overnight at 4 °C. The next day, the plates were washed five times with PBS-T and blocked with 5% PBS-skimmed milk (PBS-SM) for 1 h at 37 °C. After washing with PBS-T, duplicate serum samples were diluted 1:250 in PBS-SM, and 50 μl of diluted serum were added to each well. The plates were incubated at 37 °C for 1 h and washed with PBS-T five times. Specific IgG antibody was detected using horseradish-peroxidase-conjugated anti-goat IgG antibodies (Invitrogen, USA). The conjugate was diluted 1:5000 with PBS, and 50 μl of diluted conjugates were added. After incubation at 37 °C for 1 h, the plates were washed five times with PBS-T, and then 3,3′,5,5′-tetramethylbenzidine (Invitrogen, USA) was added to develop the color. After 15 min, the reaction was stopped by adding 50 μl of 0.1 M HCl. OD450 was read using a microplate reader (model ELx808, Biotex, VT, USA).

### LAT

The negative and positive control sera were confirmed using MAST® TOXOREAGENT (Mast Group, Liverpool, UK). Positive samples were considered when agglutination was observed at a dilution of 1:32 or greater.

### Statistical analyses

The results of iELISA and LAT were calculated using online software (http://vassarstats.net) to determine the percentage of agreement, sensitivity, specificity, and the kappa values with 95% confidence intervals. The strength of agreement was graded as fair (κ = 0.21–0.40), moderate (κ = 0.41–0.60), and substantial (κ =0.61–0.80).

## Supplementary Information

**Additional file 1.**

**Additional file 2.**

## Data Availability

The datasets used and/or analyzed during the current study are available from the corresponding author on reasonable request.

## Abbreviation

ELISA: Enzyme-linked immunosorbent assay
LAT: Latex agglutination test
PBS: Phosphate-buffered saline
PV: Parasitophorous vacuole
