# Supplementary figures and images for: Development and evaluation of recombinant GRA8 protein for the serodiagnosis of Toxoplasma gondii infection in goats
Source: BMC Vet Res. 2021 Jan 9;17:27. doi: 10.1186/s12917-020-02719-3 (PMC7796619; doi:10.1186/s12917-020-02719-3)

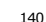

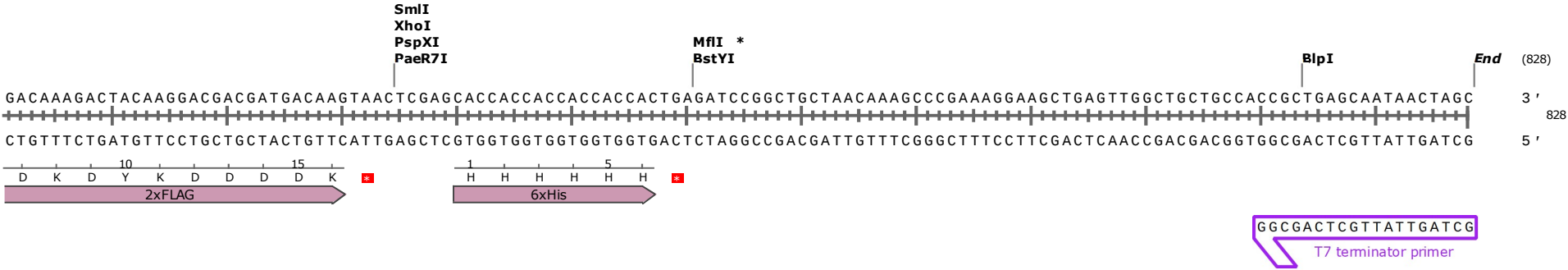

Supplement: Supplementary file 1 — Additional file 1. [file 12917_2020_2719_MOESM1_ESM.pdf]
