# Supplementary material for: Development and evaluation of recombinant GRA8 protein for the serodiagnosis of Toxoplasma gondii infection in goats
Source: BMC Vet Res. 2021 Jan 9;17:27. doi: 10.1186/s12917-020-02719-3 (PMC7796619; doi:10.1186/s12917-020-02719-3)
Supplement: Supplementary file 2 — Additional file 2. [file 12917_2020_2719_MOESM2_ESM.pdf]

MATRIX SCIENCE MASCOT Search Results

Protein View: GRA8

GRA8

**Database:**	GRA8
**Score:**	5552
**Nominal mass (M_r):**	20296
**Calculated pI:**	9.75

Sequence similarity is available as **an NCBI BLAST search of GRA8 against nr**.

Search parameters

**MS data file:**	recombinant-protein.mgf
**Enzyme:**	No enzyme cleavage specificity.
**Fixed modifications:**	**Carbamidomethyl (C)**
**Variable modifications:**	**Oxidation (M)**

Protein sequence coverage: 98%

Matched peptides shown in ***bold red***.

```
  1 MNGPLSYHPS SYGASYPNPS NPLHGMPKPE NPVRPPPPGF HPSVIPNPPY
 51 PLGTPAGMPQ PEVPPLQHPP PTGSPPAAAP QPPYPVGTPG MPQPEIPPVH
101 RPPPPGFRPE VAPVPPYPVG TPTGMPQPEI PAVHHPFPYV TTTTTAAPRV
151 LVYKIPYGGA APPRAPPVPP RMGPSDISTH VRGAIRRQPA TATT
```

Unformatted sequence string: **194 residues** (for pasting into other applications).

Sort peptides by ◉ Residue Number ○ Increasing Mass ○ Decreasing Mass

Query	Start – End	Observed	Mr(expt)	Mr(calc)	Delta	M	Score	Expect	Rank	U	Peptide
920	1 – 9	508.2422	1014.4698	1014.4593	0.0105	0	2	0.61	1	U	-.MNGPLSYHP.S
1483	1 – 12	677.2751	1352.5357	1351.5867	0.9490	0	13	0.048	1	U	-.MNGPLSYHPSSY.G
2030	1 – 18	971.3982	1940.7819	1940.8363	-0.0544	0	14	0.044	1	U	-.MNGPLSYHPSSYGASYPN.P
2031	1 – 18	971.8852	1941.7558	1940.8363	0.9196	0	16	0.028	1	U	-.MNGPLSYHPSSYGASYPN.P
1960	2 – 18	905.8770	1809.7394	1809.7958	-0.0564	0	26	0.0028	1	U	M.NGPLSYHPSSYGASYPN.P
1961	2 – 18	906.3697	1810.7249	1809.7958	0.9291	0	10	0.11	1	U	M.NGPLSYHPSSYGASYPN.P
2109	2 – 21	1054.9343	2107.8539	2107.9235	-0.0696	0	33	0.00052	1	U	M.NGPLSYHPSSYGASYPNPSN.P
2110	2 – 21	1055.4364	2108.8583	2107.9235	0.9347	0	33	0.00046	1	U	M.NGPLSYHPSSYGASYPNPSN.P
2615	2 – 31	803.0994	3208.3687	3208.4672	-0.0986	0	12	0.062	1	U	M.NGPLSYHPSSYGASYPNPSNPLHGMPKPEN.P
2092	3 – 22	523.7709	2091.0544	2090.9334	0.1211	0	0	0.99	1	U	N.GPLSYHPSSYGASYPNPSNP.L
2280	5 – 26	797.7121	2390.1145	2391.0590	-0.9445	0	4	0.44	1	U	P.LSYHPSSYGASYPNPSNPLHGM.P + Oxidation (M)
765	6 – 13	300.1242	897.3508	896.3664	0.9843	0	1	0.81	1	U	L.SYHPSSYG.A
769	6 – 13	300.1261	897.3566	896.3664	0.9901	0	9	0.13	1	U	L.SYHPSSYG.A
1805	7 – 21	820.8305	1639.6465	1639.6903	-0.0438	0	12	0.067	1	U	S.YHPSSYGASYPNPSN.P
1851	9 – 24	563.2581	1686.7524	1686.7638	-0.0113	0	0	0.91	1	U	H.PSSYGASYPNPSNPLH.G
2115	9 – 28	706.0026	2114.9859	2115.9684	-0.9825	0	3	0.47	1	U	H.PSSYGASYPNPSNPLHGMPK.P + Oxidation (M)
2117	9 – 28	706.3389	2115.9949	2115.9684	0.0265	0	3	0.54	1	U	H.PSSYGASYPNPSNPLHGMPK.P + Oxidation (M)
2369	9 – 32	508.5972	2537.9494	2537.1645	0.7849	0	5	0.32	1	U	H.PSSYGASYPNPSNPLHGMPKPENP.V
1306	10 – 21	621.8133	1241.6120	1242.5153	-0.9033	0	6	0.25	1	U	P.SSYGASYPNPSN.P
1855	11 – 26	423.7494	1690.9686	1690.7409	0.2277	0	8	0.15	1	U	S.SYGASYPNPSNPLHGM.P
1939	11 – 27	448.1959	1788.7546	1787.7937	0.9609	0	3	0.45	1	U	S.SYGASYPNPSNPLHGMP.K
2071	11 – 29	677.6909	2030.0509	2028.9363	1.1146	0	2	0.66	1	U	S.SYGASYPNPSNPLHGMPKP.E + Oxidation (M)
998	12 – 21	534.7748	1067.5350	1068.4512	-0.9162	0	5	0.3	1	U	S.YGASYPNPSN.P
2450	12 – 36	680.0392	2716.1278	2715.3227	0.8051	0	4	0.37	1	U	S.YGASYPNPSNPLHGMPKPENPVRPP.P
909	13 – 22	501.7695	1001.5245	1002.4407	-0.9162	0	0	0.98	1	U	Y.GASYPNPSNP.L
2032	14 – 31	650.6258	1948.8556	1948.9101	-0.0545	0	21	0.0073	1	U	G.ASYPNPSNPLHGMPKPEN.P
1559	15 – 27	470.8917	1409.6534	1409.6398	0.0136	0	1	0.76	1	U	A.SYPNPSNPLHGMP.K
2091	15 – 33	697.9613	2090.8620	2089.9891	0.8729	0	0	0.99	1	U	A.SYPNPSNPLHGMPKPENPV.R + Oxidation (M)
2240	15 – 35	388.7008	2326.1611	2327.1481	-0.9869	0	2	0.66	1	U	A.SYPNPSNPLHGMPKPENPVRP.P
2248	15 – 35	587.0689	2344.2465	2343.1430	1.1035	0	4	0.41	1	U	A.SYPNPSNPLHGMPKPENPVRP.P + Oxidation (M)
2495	17 – 43	579.8687	2894.3071	2893.4446	0.8625	0	0	0.95	1	U	Y.PNPSNPLHGMPKPENPVRPPPPGFHPS.V
1578	18 – 30	472.9128	1415.7166	1416.6820	-0.9654	0	3	0.46	1	U	P.NPSNPLHGMPKPE.N
851	19 – 27	482.7557	963.4969	964.4436	-0.9468	0	23	0.0045	1	U	N.PSNPLHGMP.K + Oxidation (M)
1447	19 – 30	440.2392	1317.6957	1318.6340	-0.9383	0	5	0.31	1	U	N.PSNPLHGMPKPE.N + Oxidation (M)
2635	20 – 51	435.9431	3479.4864	3478.7608	0.7255	0	4	0.36	1	U	P.SNPLHGMPKPENPVRPPPPGFHPSVIPNPPYP.L + Oxidation (M)
1861	21 – 35	567.2698	1698.7877	1697.8671	0.9205	0	7	0.2	1	U	S.NPLHGMPKPENPVRP.P + Oxidation (M)
2171	23 – 42	555.3031	2217.1833	2216.1313	1.0519	0	1	0.8	1	U	P.LHGMPKPENPVRPPPPGFHP.S + Oxidation (M)
2329	24 – 46	828.7529	2483.2369	2483.2896	-0.0527	0	4	0.42	1	U	L.HGMPKPENPVRPPPPGFHPSVIP.N
1690	25 – 38	765.4053	1528.7960	1527.7868	1.0093	0	4	0.38	1	U	H.GMPKPENPVRPPPP.G + Oxidation (M)
1691	25 – 38	765.4072	1528.7998	1527.7868	1.0131	0	3	0.54	1	U	H.GMPKPENPVRPPPP.G + Oxidation (M)
1696	25 – 38	510.6088	1528.8045	1527.7868	1.0178	0	3	0.47	1	U	H.GMPKPENPVRPPPP.G + Oxidation (M)
2077	25 – 43	679.9942	2036.9608	2037.0255	-0.0646	0	6	0.23	1	U	H.GMPKPENPVRPPPPGFHPS.V
1023	26 – 34	361.5282	1081.5629	1082.5542	-0.9914	0	11	0.073	1	U	G.MPKPENPVR.P + Oxidation (M)
2155	26 – 45	1104.9050	2207.7955	2208.1514	-0.3559	0	2	0.69	1	U	G.MPKPENPVRPPPPGFHPSVI.P + Oxidation (M)

Query	Start – End	Observed	Mr(expt)	Mr(calc)	Delta	M	Score	Expect	Rank	U	Peptide
2640	26 – 58	438.7285	3501.7699	3500.7737	0.9961	0	1	0.82	1	U	G.MPKPENPVRPPPPGFHPSVIPNPPYPLGTPAGM.P + Oxidation (M)
2131	29 – 48	715.6927	2144.0563	2144.1167	-0.0604	0	4	0.44	1	U	K.PENPVRPPPPGFHPSVIPNP.P
2132	29 – 48	715.6934	2144.0584	2144.1167	-0.0583	0	7	0.22	1	U	K.PENPVRPPPPGFHPSVIPNP.P
626	30 – 36	405.1836	808.3527	807.4239	0.9288	0	24	0.0038	1	U	P.ENPVRPP.P
2400	32 – 56	650.9772	2599.8796	2600.3904	-0.5108	0	0	0.92	1	U	N.PVRPPPPGFHPSVIPNPPYPLGTPA.G
2477	32 – 58	930.8874	2789.6403	2788.4523	1.1880	0	4	0.39	1	U	N.PVRPPPPGFHPSVIPNPPYPLGTPAGM.P
565	35 – 41	375.1810	748.3475	747.3704	0.9771	0	4	0.39	1	U	R.PPPPGFH.P
2106	40 – 59	701.9919	2102.9538	2104.0452	-1.0914	0	3	0.55	1	U	G.FHPSVIPNPPYPLGTPAGMP.Q + Oxidation (M)
1817	41 – 56	829.4453	1656.8760	1655.8671	1.0089	0	0	0.97	1	U	F.HPSVIPNPPYPLGTPA.G
1746	42 – 57	788.3444	1574.6743	1575.8297	-1.1554	0	2	0.63	1	U	H.PSVIPNPPYPLGTPAG.M
2618	42 – 72	1075.0660	3222.1763	3221.6584	0.5179	0	1	0.72	1	U	H.PSVIPNPPYPLGTPAGMPQPEVPPLQHPPPT.G
1983	43 – 60	617.9596	1850.8571	1850.9237	-0.0666	0	0	0.93	1	U	P.SVIPNPPYPLGTPAGMPQ.P + Oxidation (M)
2332	43 – 66	497.6558	2483.2426	2483.2770	-0.0344	0	1	0.85	1	U	P.SVIPNPPYPLGTPAGMPQPEVPPL.Q + Oxidation (M)
2458	43 – 68	911.9396	2732.7969	2732.3996	0.3973	0	5	0.3	1	U	P.SVIPNPPYPLGTPAGMPQPEVPPLQH.P
1532	44 – 57	697.3504	1392.6861	1391.7449	0.9412	0	0	0.94	1	U	S.VIPNPPYPLGTPAG.M
1603	45 – 58	480.9538	1439.8397	1439.7119	0.1278	0	4	0.44	1	U	V.IPNPPYPLGTPAGM.P + Oxidation (M)
1992	47 – 64	626.2992	1875.8757	1876.9029	-1.0272	0	0	0.9	1	U	P.NPPYPLGTPAGMPQPEVP.P + Oxidation (M)
1830	48 – 63	833.8398	1665.6650	1665.8073	-0.1423	0	3	0.54	1	U	N.PPYPLGTPAGMPQPEV.P + Oxidation (M)
1051	49 – 59	550.2428	1098.4710	1099.5372	-1.0662	0	1	0.81	1	U	P.PYPLGTPAGMP.Q
1052	49 – 59	550.2454	1098.4763	1099.5372	-1.0609	0	1	0.78	1	U	P.PYPLGTPAGMP.Q
1474	49 – 61	671.3183	1340.6221	1340.6435	-0.0214	0	1	0.77	1	U	P.PYPLGTPAGMPQP.E + Oxidation (M)
1026	51 – 61	541.8312	1081.6478	1080.5274	1.1204	0	0	0.98	1	U	Y.PLGTPAGMPQP.E + Oxidation (M)
2244	51 – 73	583.5715	2330.2567	2330.1729	0.0838	0	6	0.25	1	U	Y.PLGTPAGMPQPEVPPLQHPPPTG.S + Oxidation (M)
459	52 – 58	331.1883	660.3621	661.3105	-0.9484	0	4	0.4	1	U	P.LGTPAGM.P + Oxidation (M)
575	52 – 59	380.7144	759.4143	758.3633	1.0510	0	2	0.57	1	U	P.LGTPAGMP.Q + Oxidation (M)
1231	52 – 63	606.3722	1210.7297	1211.5856	-0.8559	0	0	0.95	1	U	P.LGTPAGMPQPEV.P + Oxidation (M)
589	53 – 60	388.1821	774.3495	773.3378	1.0117	0	4	0.42	1	U	L.GTPAGMPQ.P + Oxidation (M)
2011	54 – 71	953.4355	1904.8564	1904.9455	-0.0891	0	1	0.78	1	U	G.TPAGMPQPEVPPLQHPPP.T + Oxidation (M)
2139	54 – 74	538.7933	2151.1441	2150.0467	1.0974	0	0	0.9	1	U	G.TPAGMPQPEVPPLQHPPPTGS.P + Oxidation (M)
1285	55 – 66	411.8663	1232.5772	1231.6271	0.9501	0	6	0.28	1	U	T.PAGMPQPEVPPL.Q
1955	55 – 71	601.9499	1802.8278	1803.8978	-1.0700	0	5	0.33	1	U	T.PAGMPQPEVPPLQHPPP.T + Oxidation (M)
1956	55 – 71	602.2891	1803.8455	1803.8978	-0.0523	0	2	0.7	1	U	T.PAGMPQPEVPPLQHPPP.T + Oxidation (M)
819	56 – 64	470.7381	939.4616	940.4324	-0.9708	0	1	0.79	1	U	P.AGMPQPEVP.P + Oxidation (M)
1362	56 – 67	421.8828	1262.6266	1262.6329	-0.0063	0	7	0.19	1	U	P.AGMPQPEVPPLQ.H
1892	58 – 73	431.4597	1721.8099	1720.8607	0.9492	0	3	0.45	1	U	G.MPQPEVPPLQHPPPTG.S
2366	61 – 85	508.5962	2537.9447	2538.2907	-0.3460	0	1	0.75	1	U	Q.PEVPPLQHPPPTGSPPAAAPQPPYP.V
717	65 – 77	432.2681	1293.7826	1294.6670	-0.8844	0	2	0.66	1	U	P.PLQHPPPTGSPPA.A
1601	65 – 79	719.8013	1437.5880	1436.7412	0.8468	0	2	0.68	1	U	P.PLQHPPPTGSPPAAA.P
2620	66 – 97	463.7392	3239.1232	3238.6121	0.5111	0	1	0.73	1	U	P.LQHPPPTGSPPAAAPQPPYPVGTPGMPQPEIP.P + Oxidation (M)
2014	72 – 91	954.4344	1906.8543	1907.9088	-1.0545	0	0	0.91	1	U	P.TGSPPAAAPQPPYPVGTPGM.P + Oxidation (M)
1580	76 – 90	473.9140	1418.7201	1418.7194	0.0007	0	3	0.51	1	U	P.PAAAPQPPYPVGTPG.M
1940	77 – 94	895.8966	1789.7787	1790.8662	-1.0875	0	4	0.43	1	U	P.AAAPQPPYPVGTPGMPQP.E + Oxidation (M)
2012	77 – 95	635.9601	1904.8586	1903.9138	0.9447	0	8	0.17	1	U	P.AAAPQPPYPVGTPGMPQPE.I
2314	77 – 100	822.4660	2464.3761	2463.2257	1.1505	0	3	0.55	1	U	P.AAAPQPPYPVGTPGMPQPEIPPVH.R + Oxidation (M)
1324	78 – 90	625.7329	1249.4513	1250.6295	-1.1782	0	4	0.43	1	U	A.AAPQPPYPVGTPG.M
1891	78 – 94	574.2850	1719.8331	1719.8291	0.0040	0	0	1	1	U	A.AAPQPPYPVGTPGMPQP.E + Oxidation (M)
2342	81 – 103	835.3742	2503.1007	2503.2682	-0.1675	0	0	0.94	1	U	P.QPPYPVGTPGMPQPEIPPVHRPP.P + Oxidation (M)
1130	83 – 93	579.7897	1157.5648	1158.5380	-0.9732	0	2	0.59	1	U	P.PYPVGTPGMPQ.P + Oxidation (M)
1294	83 – 94	413.8658	1238.5756	1239.5958	-1.0202	0	6	0.25	1	U	P.PYPVGTPGMPQP.E
1515	83 – 95	692.8509	1383.6872	1384.6333	-0.9461	0	1	0.75	1	U	P.PYPVGTPGMPQPE.I + Oxidation (M)
1656	84 – 97	375.6397	1498.5298	1497.7174	0.8124	0	5	0.31	1	U	P.YPVGTPGMPQPEIP.P + Oxidation (M)
2270	84 – 105	594.7448	2374.9501	2375.2096	-0.2596	0	1	0.81	1	U	P.YPVGTPGMPQPEIPPVHRPPPP.G + Oxidation (M)
621	86 – 93	402.1994	802.3842	801.3691	1.0151	0	5	0.34	1	U	P.VGTPGMPQ.P + Oxidation (M)
766	86 – 94	300.1252	897.3537	898.4219	-1.0682	0	2	0.67	1	U	P.VGTPGMPQP.E + Oxidation (M)
1597	86 – 99	717.8404	1433.6662	1433.7225	-0.0563	0	1	0.78	1	U	P.VGTPGMPQPEIPPV.H + Oxidation (M)
2277	91 – 111	598.2258	2388.8742	2389.2365	-0.3623	0	5	0.32	1	U	G.MPQPEIPPVHRPPPPGFRPEV.A + Oxidation (M)
1148	93 – 102	390.2054	1167.5945	1168.6353	-1.0408	0	6	0.28	1	U	P.QPEIPPVHRP.P
1676	93 – 106	759.3780	1516.7414	1516.8150	-0.0736	0	1	0.75	1	U	P.QPEIPPVHRPPPPG.F
1287	95 – 105	617.8261	1233.6376	1234.6822	-1.0446	0	5	0.35	1	U	P.EIPPVHRPPPP.G
2652	98 – 132	308.9090	3694.8208	3693.9130	0.9078	0	3	0.54	1	U	P.PVHRPPPPGFRPEVAPVPPYPVGTPTGMPQPEIPA.V + Oxidation (M)
1144	102 – 112	582.3066	1162.5986	1162.6135	-0.0149	0	2	0.59	1	U	R.PPPPGFRPEVA.P
1138	103 – 113	582.2905	1162.5664	1162.6135	-0.0471	0	1	0.85	1	U	P.PPPGFRPEVAP.V
1747	109 – 124	788.8821	1575.7495	1576.8137	-1.0642	0	3	0.48	1	U	R.PEVAPVPPYPVGTPTG.M
2487	109 – 135	944.4320	2830.2740	2829.4160	0.8580	0	5	0.33	1	U	R.PEVAPVPPYPVGTPTGMPQPEIPAVHH.P + Oxidation (M)
2087	114 – 133	516.2721	2061.0593	2062.0445	-0.9852	0	8	0.16	1	U	P.VPPYPVGTPTGMPQPEIPAV.H + Oxidation (M)
1981	115 – 132	308.9911	1847.9031	1847.9128	-0.0097	0	5	0.3	1	U	V.PPYPVGTPTGMPQPEIPA.V
1473	116 – 128	671.3183	1340.6221	1340.6435	-0.0214	0	8	0.15	1	U	P.PYPVGTPTGMPQP.E
1996	118 – 135	940.9409	1879.8673	1879.9251	-0.0578	0	3	0.47	1	U	Y.PVGTPTGMPQPEIPAVHH.P + Oxidation (M)
2120	118 – 137	425.8540	2124.2338	2124.0463	0.1875	0	2	0.68	1	U	Y.PVGTPTGMPQPEIPAVHHPF.P + Oxidation (M)
773	120 – 128	451.2126	900.4107	900.4011	0.0095	0	6	0.26	1	U	V.GTPTGMPQP.E + Oxidation (M)
2510	120 – 147	734.7874	2935.1205	2934.4222	0.6983	0	3	0.47	1	U	V.GTPTGMPQPEIPAVHHPFPYVTTTTTAA.P + Oxidation (M)
1000	121 – 130	536.2042	1070.3937	1069.5114	0.8823	0	2	0.7	1	U	G.TPTGMPQPEI.P

Query	Start – End	Observed	Mr(expt)	Mr(calc)	Delta	M	Score	Expect	Rank	U	Peptide
2593	121 – 149	348.8182	3130.2984	3130.5546	-0.2562	0	2	0.59	1	U	G.TPTGMPQPEIPAVHHPFPYVTTTTTAAPR.V + Oxidation (M)
1019	122 – 131	541.2808	1080.5471	1081.5114	-0.9643	0	2	0.65	1	U	T.PTGMPQPEIP.A + Oxidation (M)
2123	122 – 140	711.0158	2130.0255	2129.0405	0.9850	0	0	0.91	1	U	T.PTGMPQPEIPAVHHPFPYV.T + Oxidation (M)
1566	123 – 135	471.8595	1412.5567	1412.6871	-0.1303	0	0	0.91	1	U	P.TGMPQPEIPAVHH.P
1971	124 – 139	916.9243	1831.8341	1831.8716	-0.0375	0	1	0.71	1	U	T.GMPQPEIPAVHHPFPY.V + Oxidation (M)
2205	125 – 144	570.7076	2278.8013	2278.1093	0.6920	0	3	0.54	1	U	G.MPQPEIPAVHHPFPYVTTTT.T + Oxidation (M)
2125	126 – 144	533.5139	2130.0264	2131.0739	-1.0475	0	9	0.12	1	U	M.PQPEIPAVHHPFPYVTTTT.T
2412	126 – 149	876.7603	2627.2590	2627.3496	-0.0907	0	25	0.0035	1	U	M.PQPEIPAVHHPFPYVTTTTTAAPR.V
2413	126 – 149	657.8233	2627.2642	2627.3496	-0.0854	0	24	0.0038	1	U	M.PQPEIPAVHHPFPYVTTTTTAAPR.V
2414	126 – 149	876.7621	2627.2646	2627.3496	-0.0851	0	39	0.00014	1	U	M.PQPEIPAVHHPFPYVTTTTTAAPR.V
2415	126 – 149	657.8237	2627.2657	2627.3496	-0.0839	0	42	6.6e-005	1	U	M.PQPEIPAVHHPFPYVTTTTTAAPR.V
2416	126 – 149	657.8251	2627.2712	2627.3496	-0.0784	0	42	6e-005	1	U	M.PQPEIPAVHHPFPYVTTTTTAAPR.V
1800	127 – 140	544.5920	1630.7542	1629.8304	0.9238	0	0	0.95	1	U	P.QPEIPAVHHPFPYV.T
2041	132 – 149	655.9654	1964.8744	1966.0061	-1.1317	0	35	0.00034	1	U	P.AVHHPFPYVTTTTTAAPR.V
1662	133 – 145	751.4011	1500.7877	1499.7409	1.0468	0	12	0.058	1	U	A.VHHPFPYVTTTTT.A
2003	133 – 149	948.4594	1894.9042	1894.9690	-0.0648	0	8	0.16	1	U	A.VHHPFPYVTTTTTAAPR.V
2005	133 – 149	632.6468	1894.9186	1894.9690	-0.0504	0	44	3.8e-005	1	U	A.VHHPFPYVTTTTTAAPR.V
1823	135 – 149	830.4028	1658.7910	1658.8417	-0.0506	0	17	0.02	1	U	H.HPFPYVTTTTTAAPR.V
1824	135 – 149	553.9400	1658.7982	1658.8417	-0.0435	0	51	7.3e-006	1	U	H.HPFPYVTTTTTAAPR.V
1681	136 – 149	761.8774	1521.7403	1521.7828	-0.0425	0	45	3.4e-005	1	U	H.PFPYVTTTTTAAPR.V
1586	137 – 149	713.3512	1424.6878	1424.7300	-0.0421	0	63	4.8e-007	1	U	P.FPYVTTTTTAAPR.V
1079	138 – 148	561.7635	1121.5125	1121.5605	-0.0480	0	3	0.47	1	U	F.PYVTTTTTAAP.R
1166	139 – 149	591.2961	1180.5777	1180.6088	-0.0311	0	22	0.0058	1	U	P.YVTTTTTAAPR.V
1167	139 – 149	591.2963	1180.5781	1180.6088	-0.0307	0	47	1.9e-005	1	U	P.YVTTTTTAAPR.V
2013	142 – 159	636.3260	1905.9561	1907.0517	-1.0956	0	0	0.94	1	U	T.TTTTAAPRVLVYKIPYGG.A
2152	144 – 164	1099.0323	2196.0500	2197.2371	-1.1871	0	10	0.11	1	U	T.TTAAPRVLVYKIPYGGAAPPR.A
2658	148 – 182	937.9980	3747.9631	3748.0511	-0.0881	0	4	0.43	1	U	A.PRVLVYKIPYGGAAPPRAPPVPPRMGPSDISTHVR.G
1165	150 – 160	590.3268	1178.6390	1178.6699	-0.0309	0	23	0.0056	1	U	R.VLVYKIPYGGA.A
1768	150 – 164	800.9394	1599.8643	1599.9137	-0.0494	0	56	2.6e-006	1	U	R.VLVYKIPYGGAAPPR.A
1769	150 – 164	534.2959	1599.8659	1599.9137	-0.0478	0	69	1.3e-007	1	U	R.VLVYKIPYGGAAPPR.A
1770	150 – 164	534.2960	1599.8662	1599.9137	-0.0475	0	25	0.0035	1	U	R.VLVYKIPYGGAAPPR.A
1771	150 – 164	800.9405	1599.8663	1599.9137	-0.0473	0	71	7.5e-008	1	U	R.VLVYKIPYGGAAPPR.A
1772	150 – 164	534.2962	1599.8667	1599.9137	-0.0470	0	61	7.7e-007	1	U	R.VLVYKIPYGGAAPPR.A
1773	150 – 164	534.2962	1599.8667	1599.9137	-0.0470	0	69	1.3e-007	1	U	R.VLVYKIPYGGAAPPR.A
1774	150 – 164	534.2963	1599.8670	1599.9137	-0.0467	0	56	2.3e-006	1	U	R.VLVYKIPYGGAAPPR.A
1775	150 – 164	534.2966	1599.8681	1599.9137	-0.0456	0	30	0.001	1	U	R.VLVYKIPYGGAAPPR.A
1776	150 – 164	534.2967	1599.8682	1599.9137	-0.0455	0	45	3.5e-005	1	U	R.VLVYKIPYGGAAPPR.A
1777	150 – 164	534.2967	1599.8683	1599.9137	-0.0454	0	39	0.00012	1	U	R.VLVYKIPYGGAAPPR.A
1778	150 – 164	534.2967	1599.8683	1599.9137	-0.0454	0	33	0.00046	1	U	R.VLVYKIPYGGAAPPR.A
2232	150 – 171	579.5722	2314.2595	2314.3314	-0.0718	0	51	8.8e-006	1	U	R.VLVYKIPYGGAAPPRAPPVPPR.M
2233	150 – 171	579.5722	2314.2598	2314.3314	-0.0716	0	75	3.2e-008	1	U	R.VLVYKIPYGGAAPPRAPPVPPR.M
2234	150 – 171	772.4285	2314.2636	2314.3314	-0.0678	0	29	0.0012	1	U	R.VLVYKIPYGGAAPPRAPPVPPR.M
1663	151 – 164	501.2715	1500.7926	1500.8453	-0.0527	0	34	0.00045	1	U	V.LVYKIPYGGAAPPR.A
1664	151 – 164	501.2748	1500.8026	1500.8453	-0.0427	0	35	0.00032	1	U	V.LVYKIPYGGAAPPR.A
1519	152 – 164	694.8683	1387.7221	1387.7612	-0.0391	0	58	1.7e-006	1	U	L.VYKIPYGGAAPPR.A
1520	152 – 164	463.5818	1387.7235	1387.7612	-0.0377	0	68	1.6e-007	1	U	L.VYKIPYGGAAPPR.A
1521	152 – 164	694.8691	1387.7236	1387.7612	-0.0376	0	50	9.8e-006	1	U	L.VYKIPYGGAAPPR.A
1522	152 – 164	463.5849	1387.7327	1387.7612	-0.0285	0	40	9.6e-005	1	U	L.VYKIPYGGAAPPR.A
2104	152 – 171	701.7120	2102.1140	2102.1789	-0.0649	0	24	0.0042	1	U	L.VYKIPYGGAAPPRAPPVPPR.M
2105	152 – 171	526.5376	2102.1214	2102.1789	-0.0575	0	26	0.0026	1	U	L.VYKIPYGGAAPPRAPPVPPR.M
2643	152 – 185	504.2675	3522.8214	3523.8874	-1.0661	0	0	0.97	1	U	L.VYKIPYGGAAPPRAPPVPPRMGPSDISTHVRGAI.R
1410	153 – 164	430.2295	1287.6668	1288.6928	-1.0260	0	15	0.035	1	U	V.YKIPYGGAAPPR.A
1412	153 – 164	645.3320	1288.6495	1288.6928	-0.0433	0	21	0.0076	1	U	V.YKIPYGGAAPPR.A
2160	153 – 173	442.5681	2207.8042	2207.1673	0.6369	0	2	0.67	1	U	V.YKIPYGGAAPPRAPPVPPRMG.P + Oxidation (M)
1329	153 – 176	627.3524	2505.3804	2506.2791	-0.8987	0	3	0.61	1	U	V.YKIPYGGAAPPRAPPVPPRMGPSD.I + Oxidation (M)
1081	154 – 164	563.2944	1124.5743	1125.6295	-1.0552	0	8	0.17	1	U	Y.KIPYGGAAPPR.A
1084	154 – 164	563.8069	1125.5991	1125.6295	-0.0303	0	66	2.8e-007	1	U	Y.KIPYGGAAPPR.A
1085	154 – 164	376.2079	1125.6019	1125.6295	-0.0276	0	38	0.00016	1	U	Y.KIPYGGAAPPR.A
1973	154 – 171	461.0067	1839.9978	1840.0472	-0.0493	0	26	0.0027	1	U	Y.KIPYGGAAPPRAPPVPPR.M
892	155 – 164	499.7609	997.5073	997.5345	-0.0272	0	70	9.7e-008	1	U	K.IPYGGAAPPR.A
893	155 – 164	499.7610	997.5075	997.5345	-0.0270	0	65	3.5e-007	1	U	K.IPYGGAAPPR.A
894	155 – 164	499.7615	997.5084	997.5345	-0.0261	0	64	3.6e-007	1	U	K.IPYGGAAPPR.A
895	155 – 164	499.7616	997.5085	997.5345	-0.0260	0	70	1.1e-007	1	U	K.IPYGGAAPPR.A
896	155 – 164	499.7616	997.5086	997.5345	-0.0259	0	62	5.6e-007	1	U	K.IPYGGAAPPR.A
897	155 – 164	499.7616	997.5086	997.5345	-0.0259	0	26	0.0026	1	U	K.IPYGGAAPPR.A
898	155 – 164	499.7616	997.5087	997.5345	-0.0258	0	34	0.00035	1	U	K.IPYGGAAPPR.A
899	155 – 164	499.7617	997.5088	997.5345	-0.0257	0	68	1.7e-007	1	U	K.IPYGGAAPPR.A
900	155 – 164	499.7618	997.5089	997.5345	-0.0256	0	67	1.9e-007	1	U	K.IPYGGAAPPR.A
901	155 – 164	499.7621	997.5096	997.5345	-0.0249	0	70	9.6e-008	1	U	K.IPYGGAAPPR.A
902	155 – 164	499.7622	997.5098	997.5345	-0.0247	0	20	0.0093	1	U	K.IPYGGAAPPR.A
903	155 – 164	499.7630	997.5114	997.5345	-0.0231	0	67	2.1e-007	1	U	K.IPYGGAAPPR.A
904	155 – 164	499.7635	997.5125	997.5345	-0.0220	0	63	5e-007	1	U	K.IPYGGAAPPR.A
905	155 – 164	499.7635	997.5125	997.5345	-0.0220	0	19	0.013	1	U	K.IPYGGAAPPR.A
906	155 – 164	499.7636	997.5126	997.5345	-0.0219	0	22	0.0063	1	U	K.IPYGGAAPPR.A
907	155 – 164	500.2395	998.4644	997.5345	0.9299	0	20	0.01	1	U	K.IPYGGAAPPR.A
908	155 – 164	500.2638	998.5130	997.5345	0.9785	0	35	0.0003	1	U	K.IPYGGAAPPR.A
1876	155 – 171	571.2678	1710.7817	1711.9522	-1.1705	0	21	0.0075	1	U	K.IPYGGAAPPRAPPVPPR.M
1877	155 – 171	571.2767	1710.8082	1711.9522	-1.1440	0	0	0.99	1	U	K.IPYGGAAPPRAPPVPPR.M
1878	155 – 171	571.3049	1710.8929	1711.9522	-1.0593	0	14	0.043	1	U	K.IPYGGAAPPRAPPVPPR.M
1880	155 – 171	856.9573	1711.9001	1711.9522	-0.0521	0	21	0.008	1	U	K.IPYGGAAPPRAPPVPPR.M
1881	155 – 171	571.6410	1711.9012	1711.9522	-0.0510	0	47	2e-005	1	U	K.IPYGGAAPPRAPPVPPR.M
1882	155 – 171	571.6412	1711.9017	1711.9522	-0.0505	0	41	7.4e-005	1	U	K.IPYGGAAPPRAPPVPPR.M
1883	155 – 171	571.6417	1711.9032	1711.9522	-0.0490	0	55	3e-006	1	U	K.IPYGGAAPPRAPPVPPR.M
1884	155 – 171	571.6419	1711.9038	1711.9522	-0.0484	0	39	0.00014	1	U	K.IPYGGAAPPRAPPVPPR.M
1886	155 – 171	856.9602	1711.9058	1711.9522	-0.0464	0	18	0.017	1	U	K.IPYGGAAPPRAPPVPPR.M

Query	Start - End	Observed	Mr(expt)	Mr(calc)	Delta	M	Score	Expect	Rank	U	Peptide
2228	155 - 177	771.3538	2311.0397	2312.2099	-1.1703	0	1	0.83	1	U	K.IPYGGAAPPRAPPVPPRMGPSDI.S
749	156 - 164	443.1947	884.3749	884.4504	-0.0756	0	36	0.00026	1	U	I.PYGGAAPPR.A
750	156 - 164	443.2220	884.4294	884.4504	-0.0210	0	58	1.5e-006	1	U	I.PYGGAAPPR.A
1607	156 - 170	482.2458	1443.7157	1442.7670	0.9486	0	5	0.29	1	U	I.PYGGAAPPRAPPVPP.R
1765	156 - 171	533.9488	1598.8247	1598.8681	-0.0434	0	30	0.0011	1	U	I.PYGGAAPPRAPPVPPR.M
1766	156 - 171	533.9493	1598.8261	1598.8681	-0.0420	0	16	0.023	1	U	I.PYGGAAPPRAPPVPPR.M
1767	156 - 171	533.9505	1598.8297	1598.8681	-0.0384	0	35	0.00031	1	U	I.PYGGAAPPRAPPVPPR.M
2286	156 - 179	601.9612	2403.8158	2403.2005	0.6153	0	2	0.59	1	U	I.PYGGAAPPRAPPVPPRMGPSDIST.H + Oxidation (M)
833	157 - 166	478.2482	954.4817	955.4875	-1.0058	0	11	0.074	1	U	P.YGGAAPPRAP.P
2499	158 - 186	730.2842	2917.1076	2916.5617	0.5459	0	0	0.96	1	U	Y.GGAAPPRAPPVPPRMGPSDISTHVRGAIR.R
1396	159 - 171	641.8550	1281.6955	1281.7306	-0.0351	0	24	0.0041	1	U	G.GAAPPRAPPVPPR.M
1397	159 - 171	428.2400	1281.6981	1281.7306	-0.0325	0	34	0.00038	1	U	G.GAAPPRAPPVPPR.M
1398	159 - 171	641.8568	1281.6990	1281.7306	-0.0316	0	19	0.011	1	U	G.GAAPPRAPPVPPR.M
1399	159 - 171	428.2409	1281.7008	1281.7306	-0.0298	0	36	0.00027	1	U	G.GAAPPRAPPVPPR.M
1594	159 - 172	715.3620	1428.7095	1428.7660	-0.0564	0	2	0.65	1	U	G.GAAPPRAPPVPPRM.G + Oxidation (M)
1270	160 - 171	613.2870	1224.5595	1224.7091	-0.1496	0	7	0.21	1	U	G.AAPPRAPPVPPR.M
1274	160 - 171	409.5677	1225.6814	1224.7091	0.9723	0	49	1.4e-005	1	U	G.AAPPRAPPVPPR.M
2187	160 - 181	755.9455	2264.8148	2265.1688	-0.3540	0	5	0.3	1	U	G.AAPPRAPPVPPRMGPSDISTHV.R + Oxidation (M)
2300	160 - 182	606.0443	2420.1481	2421.2699	-1.1218	0	1	0.8	1	U	G.AAPPRAPPVPPRMGPSDISTHVR.G + Oxidation (M)
2301	160 - 182	1211.5237	2421.0328	2421.2699	-0.2371	0	15	0.033	1	U	G.AAPPRAPPVPPRMGPSDISTHVR.G + Oxidation (M)
2302	160 - 182	808.0217	2421.0431	2421.2699	-0.2268	0	68	1.5e-007	1	U	G.AAPPRAPPVPPRMGPSDISTHVR.G + Oxidation (M)
1124	161 - 171	385.5555	1153.6448	1153.6720	-0.0272	0	46	2.6e-005	1	U	A.APPRAPPVPPR.M
1125	161 - 171	385.5559	1153.6458	1153.6720	-0.0262	0	38	0.00017	1	U	A.APPRAPPVPPR.M
1814	161 - 176	553.2737	1656.7992	1656.8406	-0.0414	0	5	0.34	1	U	A.APPRAPPVPPRMGPSD.I + Oxidation (M)
1022	162 - 171	541.7374	1081.4603	1082.6349	-1.1746	0	9	0.14	1	U	A.PPRAPPVPPR.M
1028	162 - 171	542.3089	1082.6032	1082.6349	-0.0317	0	6	0.22	1	U	A.PPRAPPVPPR.M
1858	162 - 177	850.3993	1698.7840	1698.8876	-0.1036	0	2	0.62	1	U	A.PPRAPPVPPRMGPSDI.S + Oxidation (M)
878	163 - 171	493.7687	985.5228	985.5821	-0.0594	0	3	0.55	1	U	P.PRAPPVPPR.M
880	163 - 171	494.2510	986.4874	985.5821	0.9053	0	4	0.4	1	U	P.PRAPPVPPR.M
882	163 - 171	329.8437	986.5094	985.5821	0.9273	0	8	0.16	1	U	P.PRAPPVPPR.M
1504	163 - 175	688.3355	1374.6565	1373.7238	0.9327	0	1	0.79	1	U	P.PRAPPVPPRMGPS.D + Oxidation (M)
1836	163 - 178	837.8821	1673.7495	1672.8719	0.8776	0	1	0.85	1	U	P.PRAPPVPPRMGPSDIS.T
1030	163 - 182	542.7985	2167.1648	2166.1480	1.0168	0	0	1	1	U	P.PRAPPVPPRMGPSDISTHVR.G
754	164 - 171	445.2070	888.3995	888.5294	-0.1299	0	6	0.26	1	U	P.RAPPVPPR.M
759	164 - 171	445.7298	889.4450	888.5294	0.9156	0	6	0.27	1	U	P.RAPPVPPR.M
1507	164 - 176	459.2382	1374.6927	1375.7030	-1.0104	0	1	0.77	1	U	P.RAPPVPPRMGPSD.I
532	165 - 171	367.2128	732.4110	732.4283	-0.0172	0	58	1.5e-006	1	U	R.APPVPPR.M
533	165 - 171	367.2128	732.4111	732.4283	-0.0172	0	63	5.5e-007	1	U	R.APPVPPR.M
534	165 - 171	367.2129	732.4112	732.4283	-0.0170	0	48	1.5e-005	1	U	R.APPVPPR.M
535	165 - 171	367.2129	732.4112	732.4283	-0.0170	0	57	1.9e-006	1	U	R.APPVPPR.M
536	165 - 171	367.2129	732.4113	732.4283	-0.0170	0	62	5.8e-007	1	U	R.APPVPPR.M
537	165 - 171	367.2132	732.4119	732.4283	-0.0163	0	44	4.3e-005	1	U	R.APPVPPR.M
538	165 - 171	367.2133	732.4121	732.4283	-0.0162	0	59	1.4e-006	1	U	R.APPVPPR.M
539	165 - 171	367.2138	732.4131	732.4283	-0.0152	0	30	0.0011	1	U	R.APPVPPR.M
540	165 - 171	367.2224	732.4302	732.4283	0.0020	0	59	1.3e-006	1	U	R.APPVPPR.M
1818	165 - 180	829.4453	1656.8760	1657.8246	-0.9486	0	3	0.47	1	U	R.APPVPPRMGPSDISTH.V
1145	166 - 176	582.7789	1163.5433	1164.5598	-1.0165	0	5	0.3	1	U	A.PPVPPRMGPSD.I + Oxidation (M)
1974	166 - 182	614.6735	1840.9986	1841.9570	-0.9585	0	35	0.0003	1	U	A.PPVPPRMGPSDISTHVR.G
810	167 - 175	468.7342	935.4539	936.4851	-1.0312	0	0	0.91	1	U	P.PVPPRMGPS.D
811	167 - 175	468.7344	935.4543	936.4851	-1.0308	0	0	0.9	1	U	P.PVPPRMGPS.D
812	167 - 175	468.7348	935.4550	936.4851	-1.0301	0	1	0.83	1	U	P.PVPPRMGPS.D
813	167 - 175	468.7348	935.4551	936.4851	-1.0300	0	1	0.83	1	U	P.PVPPRMGPS.D
814	167 - 175	468.7351	935.4557	936.4851	-1.0294	0	22	0.0059	1	U	P.PVPPRMGPS.D
815	167 - 175	468.7353	935.4561	936.4851	-1.0290	0	1	0.83	1	U	P.PVPPRMGPS.D
816	167 - 175	468.7356	935.4566	936.4851	-1.0286	0	13	0.054	1	U	P.PVPPRMGPS.D
2298	170 - 191	605.3042	2417.1877	2418.2662	-1.0785	0	5	0.31	1	U	P.PRMGPSDISTHVRGAIRRQPAT.A + Oxidation (M)
844	172 - 180	480.6979	959.3811	959.4019	-0.0207	0	4	0.39	1	U	R.MGPSDISTH.V + Oxidation (M)
987	172 - 181	530.7722	1059.5298	1058.4703	1.0596	0	1	0.76	1	U	R.MGPSDISTHV.R + Oxidation (M)
1191	172 - 182	1199.5470	1198.5397	1198.5765	-0.0367	0	7	0.22	1	U	R.MGPSDISTHVR.G
1192	172 - 182	600.2785	1198.5425	1198.5765	-0.0339	0	93	5.3e-010	1	U	R.MGPSDISTHVR.G
1193	172 - 182	600.2786	1198.5425	1198.5765	-0.0339	0	115	2.8e-012	1	U	R.MGPSDISTHVR.G
1194	172 - 182	600.2786	1198.5427	1198.5765	-0.0338	0	116	2.8e-012	1	U	R.MGPSDISTHVR.G
1195	172 - 182	600.2789	1198.5432	1198.5765	-0.0333	0	110	9e-012	1	U	R.MGPSDISTHVR.G
1196	172 - 182	600.2789	1198.5433	1198.5765	-0.0331	0	115	2.9e-012	1	U	R.MGPSDISTHVR.G
1197	172 - 182	400.5223	1198.5451	1198.5765	-0.0313	0	79	1.2e-008	1	U	R.MGPSDISTHVR.G
1198	172 - 182	400.5224	1198.5455	1198.5765	-0.0310	0	83	4.6e-009	1	U	R.MGPSDISTHVR.G
1199	172 - 182	400.5224	1198.5455	1198.5765	-0.0310	0	10	0.09	1	U	R.MGPSDISTHVR.G
1200	172 - 182	600.2801	1198.5456	1198.5765	-0.0309	0	102	6.8e-011	1	U	R.MGPSDISTHVR.G
1201	172 - 182	400.5225	1198.5457	1198.5765	-0.0308	0	66	2.8e-007	1	U	R.MGPSDISTHVR.G
1202	172 - 182	400.5225	1198.5458	1198.5765	-0.0307	0	22	0.0067	1	U	R.MGPSDISTHVR.G
1203	172 - 182	400.5226	1198.5458	1198.5765	-0.0306	0	80	1.1e-008	1	U	R.MGPSDISTHVR.G
1204	172 - 182	400.5228	1198.5466	1198.5765	-0.0299	0	94	4.4e-010	1	U	R.MGPSDISTHVR.G
1205	172 - 182	400.5228	1198.5466	1198.5765	-0.0299	0	94	4.3e-010	1	U	R.MGPSDISTHVR.G
1206	172 - 182	400.5231	1198.5475	1198.5765	-0.0290	0	39	0.00013	1	U	R.MGPSDISTHVR.G
1207	172 - 182	600.2819	1198.5493	1198.5765	-0.0272	0	31	0.00076	1	U	R.MGPSDISTHVR.G
1208	172 - 182	600.2836	1198.5526	1198.5765	-0.0238	0	111	8.6e-012	1	U	R.MGPSDISTHVR.G
1209	172 - 182	600.7790	1199.5435	1198.5765	0.9671	0	31	0.0008	1	U	R.MGPSDISTHVR.G
1210	172 - 182	600.8352	1199.6558	1198.5765	1.0794	0	12	0.061	1	U	R.MGPSDISTHVR.G
1211	172 - 182	600.8364	1199.6582	1198.5765	1.0817	0	2	0.68	1	U	R.MGPSDISTHVR.G
1232	172 - 182	607.8047	1213.5948	1214.5714	-0.9766	0	23	0.0053	1	U	R.MGPSDISTHVR.G + Oxidation (M)
1233	172 - 182	608.2724	1214.5303	1214.5714	-0.0411	0	82	6.2e-009	1	U	R.MGPSDISTHVR.G + Oxidation (M)
1234	172 - 182	608.2762	1214.5379	1214.5714	-0.0335	0	96	2.3e-010	1	U	R.MGPSDISTHVR.G + Oxidation (M)
1235	172 - 182	608.2763	1214.5380	1214.5714	-0.0334	0	92	5.6e-010	1	U	R.MGPSDISTHVR.G + Oxidation (M)

Query	Start – End	Observed	Mr(expt)	Mr(calc)	Delta	M	Score	Expect	Rank	U	Peptide
1236	172 – 182	608.2766	1214.5387	1214.5714	-0.0327	0	86	2.5e-009	1	U	R.MGPSDISTHVR.G + Oxidation (M)
1237	172 – 182	608.2767	1214.5388	1214.5714	-0.0326	0	93	5.4e-010	1	U	R.MGPSDISTHVR.G + Oxidation (M)
1238	172 – 182	608.2768	1214.5390	1214.5714	-0.0324	0	101	7.9e-011	1	U	R.MGPSDISTHVR.G + Oxidation (M)
1239	172 – 182	608.2769	1214.5393	1214.5714	-0.0321	0	83	4.9e-009	1	U	R.MGPSDISTHVR.G + Oxidation (M)
1240	172 – 182	405.8537	1214.5394	1214.5714	-0.0320	0	63	4.9e-007	1	U	R.MGPSDISTHVR.G + Oxidation (M)
1241	172 – 182	405.8537	1214.5394	1214.5714	-0.0320	0	62	6.5e-007	1	U	R.MGPSDISTHVR.G + Oxidation (M)
1242	172 – 182	608.2770	1214.5395	1214.5714	-0.0319	0	97	1.8e-010	1	U	R.MGPSDISTHVR.G + Oxidation (M)
1243	172 – 182	608.2771	1214.5395	1214.5714	-0.0318	0	86	2.4e-009	1	U	R.MGPSDISTHVR.G + Oxidation (M)
1244	172 – 182	608.2771	1214.5396	1214.5714	-0.0318	0	89	1.2e-009	1	U	R.MGPSDISTHVR.G + Oxidation (M)
1245	172 – 182	608.2771	1214.5396	1214.5714	-0.0317	0	106	2.8e-011	1	U	R.MGPSDISTHVR.G + Oxidation (M)
1246	172 – 182	608.2773	1214.5400	1214.5714	-0.0314	0	90	9.8e-010	1	U	R.MGPSDISTHVR.G + Oxidation (M)
1247	172 – 182	608.2773	1214.5401	1214.5714	-0.0313	0	90	9.6e-010	1	U	R.MGPSDISTHVR.G + Oxidation (M)
1248	172 – 182	405.8541	1214.5406	1214.5714	-0.0308	0	75	3.2e-008	1	U	R.MGPSDISTHVR.G + Oxidation (M)
1249	172 – 182	405.8542	1214.5408	1214.5714	-0.0305	0	45	2.9e-005	1	U	R.MGPSDISTHVR.G + Oxidation (M)
1250	172 – 182	405.8545	1214.5416	1214.5714	-0.0298	0	30	0.001	1	U	R.MGPSDISTHVR.G + Oxidation (M)
1251	172 – 182	405.8545	1214.5417	1214.5714	-0.0297	0	29	0.0013	1	U	R.MGPSDISTHVR.G + Oxidation (M)
1252	172 – 182	405.8546	1214.5419	1214.5714	-0.0294	0	79	1.2e-008	1	U	R.MGPSDISTHVR.G + Oxidation (M)
1253	172 – 182	405.8550	1214.5433	1214.5714	-0.0281	0	9	0.13	1	U	R.MGPSDISTHVR.G + Oxidation (M)
1256	172 – 182	608.2900	1214.5655	1214.5714	-0.0059	0	5	0.33	1	U	R.MGPSDISTHVR.G + Oxidation (M)
1333	172 – 183	628.2913	1254.5680	1255.5979	-1.0299	0	8	0.15	1	U	R.MGPSDISTHVRG.A
1334	172 – 183	628.2913	1254.5681	1255.5979	-1.0299	0	60	1e-006	1	U	R.MGPSDISTHVRG.A
1336	172 – 183	628.2918	1254.5691	1255.5979	-1.0289	0	41	8e-005	1	U	R.MGPSDISTHVRG.A
1337	172 – 183	628.2921	1254.5695	1255.5979	-1.0284	0	54	4.2e-006	1	U	R.MGPSDISTHVRG.A
1338	172 – 183	628.2923	1254.5700	1255.5979	-1.0279	0	60	1e-006	1	U	R.MGPSDISTHVRG.A
1339	172 – 183	628.2927	1254.5709	1255.5979	-1.0270	0	6	0.23	1	U	R.MGPSDISTHVRG.A
1346	172 – 183	419.5295	1255.5666	1255.5979	-0.0313	0	31	0.00073	1	U	R.MGPSDISTHVRG.A
1347	172 – 183	419.5298	1255.5676	1255.5979	-0.0303	0	19	0.013	1	U	R.MGPSDISTHVRG.A
1348	172 – 183	419.5300	1255.5681	1255.5979	-0.0299	0	25	0.0031	1	U	R.MGPSDISTHVRG.A
1349	172 – 183	628.7918	1255.5691	1255.5979	-0.0288	0	54	3.9e-006	1	U	R.MGPSDISTHVRG.A
1758	172 – 186	532.9318	1595.7735	1595.8202	-0.0468	0	1	0.77	1	U	R.MGPSDISTHVRGAIR.R
788	173 – 181	456.2750	910.5354	911.4349	-0.8995	0	10	0.1	1	U	M.GPSDISTHV.R
996	173 – 182	534.7619	1067.5092	1067.5360	-0.0268	0	59	1.3e-006	1	U	M.GPSDISTHVR.G
1182	173 – 184	598.2770	1194.5394	1195.5946	-1.0552	0	8	0.16	1	U	M.GPSDISTHVRGA.I
924	182 – 190	513.2436	1024.4726	1023.6050	0.8677	0	1	0.81	1	U	V.RGAIRRQPA.T

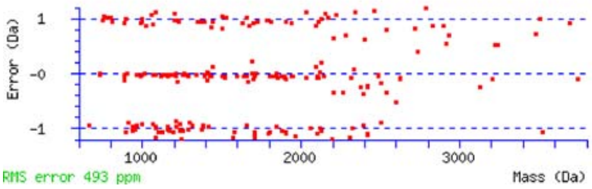

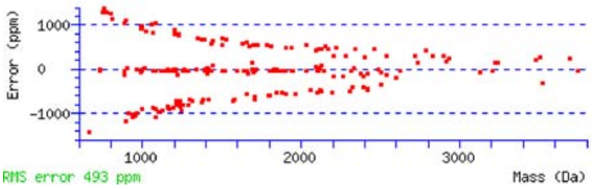

Mascot: http://www.matrixscience.com/
